# Advancements and Future Perspectives of Microfluidic Technology in Pediatric Healthcare

**DOI:** 10.1002/smmd.70018

**Published:** 2025-09-14

**Authors:** Xuting Zhang, Andong Liu, Yanke Wang, Chao Niu, Xing Rong, Chang Jia, Jia Sun, Shiyang Song, Lexiang Zhang, Fangfu Ye, Changmin Shao, Maoping Chu

**Affiliations:** ^1^ Children's Heart Center The Second Affiliated Hospital and Yuying Children's Hospital of Wenzhou Medical University Wenzhou China; ^2^ Zhejiang Provincial Clinical Research Center for Pediatric Precision Medicine Wenzhou China; ^3^ Wenzhou Institute University of Chinese Academy of Sciences Wenzhou China; ^4^ Beijing National Laboratory for Condensed Matter Physics Institute of Physics Chinese Academy of Sciences Beijing China

**Keywords:** disease diagnosis, disease modeling, disease treatments, microfluidics, pediatric healthcare

## Abstract

Due to the ability to precisely control and manipulate fluids at the microscale, microfluidics provides unmatched advantages such as reduced sample size, rapid analysis, and enhanced sensitivity. Microfluidic technology has emerged as a revolutionary approach in pediatric healthcare, offering innovative solutions for diagnostics, monitoring, and treatment. This review presents a comprehensive overview of the recent advancements and future directions of microfluidic technology in the field of pediatrics. We begin with a brief introduction of several types of microfluidic devices that are more common in the pediatric field. Then, the substantial advances in biomedical applications of microfluidics in pediatric healthcare are explored, encompassing diagnosis, research, and treatment. Finally, challenges and limitations such as material selection, device standardization, stability, and regulatory considerations are also discussed that must be addressed to increase the utilization of microfluidics in the pediatric clinical field. Overall, this review underscores the transformative potential of microfluidics to improve the quality of healthcare and outcomes for pediatric patients, while also highlighting the opportunities for future research and development in this burgeoning field.

## Key Summary

- This review provides an in‐depth examination of the recent advancements in microfluidics, focusing on its applications in pediatric diagnostics, monitoring, and treatment.
- This review discusses the key challenges and limitations, including material selection, standardization, and regulatory issues, with the aim of accelerating the clinical application of microfluidics in the pediatric field.
- This review aims to guide researchers and clinicians in effectively leveraging microfluidics to advance pediatric healthcare research and clinical practices.

## Graphical abstract

Microfluidics demonstrates significant potential to revolutionize pediatric healthcare through precise fluid manipulation, minimized sample volume and rapid, highly sensitive analysis. This review comprehensively examines recent advancements in pediatric diagnostics, monitoring and treatment, aiming to bridge research gaps and guide clinical translation.

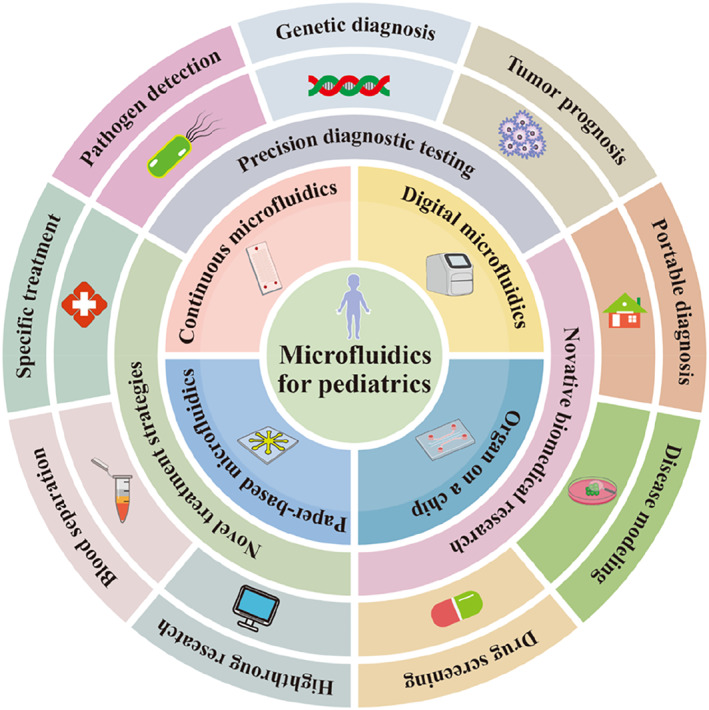

## 1 Introduction

Pediatrics, as a specialized discipline of clinical medicine, is dedicated to the healthcare of infants, children, and adolescents, addressing their unique physiological, developmental, and emotional needs. Compared with adults, children exhibit significant differences in physiology and anatomy, including drug metabolism, hormone levels, and immune responses [1, 2, 3]. With the continuous growth and development of children, children of different ages will show different reactions and pathological processes to the same pathogenic factors [4, 5, 6, 7]. In addition, the clinical manifestations of diseases in children are very different from those in adults, with congenital, hereditary and infectious diseases more common [8, 9, 10]. Although children usually have a strong ability to recover when treated promptly, these diseases are often characterized by sudden onset, unpredictable course, and a higher likelihood of complications [11, 12, 13, 14]. Because of these characteristics, the distinct characteristics of pediatrics introduce various challenges to clinical practice. First, young patients may have difficulty articulating their symptoms, resulting in inaccurate clinical information. Second, due to the small blood volume of children, careful consideration should be given to blood tests [15]. Third, pediatric treatment tends to be more conservative, with invasive procedures requiring special caution because of the unique physiological and ethical constraints of children, resulting in a relative lack of pediatric pharmacological data [16]. Finally, specific pediatric issues, such as neonatal diseases and growth‐related issues, demand tailored research approaches [17, 18]. To sum up, with the continuous development of interdisciplinary cooperation, the close integration of pediatrics with other fields is the key to promote its development.

In recent years, microfluidic technology has gained significant prominence in the medical field due to its ability to manipulate fluids at the microscopic scale [19, 20, 21]. Often referred to as a “lab‐on‐a‐chip”, a microfluidic platform is typically only a few square centimeters or less in size and integrates a variety of basic operations such as sample preparation, reaction, separation, detection, cell culture, sorting, and cleavage onto the chip [22, 23, 24]. Building upon its established utility in broader medical contexts, microfluidic technology is rapidly transforming pediatric medicine, offering unique solutions to challenges inherent in caring for children. In diagnostics, its ultra‐low sample volumes (μL) permit minimally invasive rapid point‐of‐care testing for critical conditions, reducing trauma from repeated blood draws [15]. For disease research, organ‐on‐a‐chip platforms enable ethical modeling of developing organs, supporting mechanistic studies of pediatric diseases—including developmental disorders, infections, and childhood cancers—with precise microenvironment control for drug screening [25]. Regarding treatment, microfluidics facilitate real‐time therapeutic drug monitoring, rapid antibiotic susceptibility testing, and novel cellular therapies via microfluidic cell sorting, collectively advancing personalized care while minimizing pediatric discomfort [26, 27].

**FIGURE 1:**
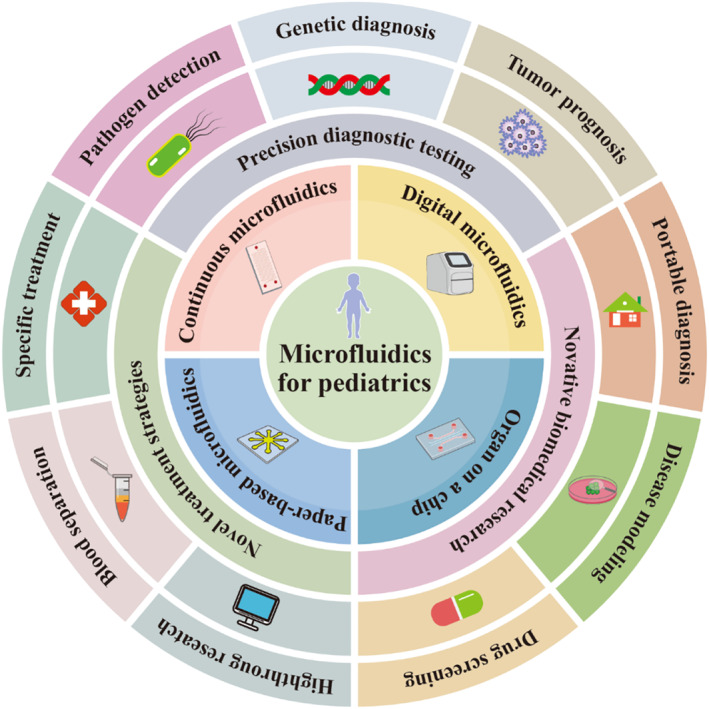
Advancements and future perspectives of microfluidic technology in pediatric healthcare.

While there have been previous reviews on microfluidics and its broad applications in biomedical fields, there remains a gap in the literature specifically focusing on the role of microfluidics in pediatric medicine [28, 29, 30]. This review aims to address this gap by providing a comprehensive analysis of microfluidics in the pediatric context, emphasizing its unique contribution and potential impact on this specialized field (Figure 1). First, the definition of microfluidic technology is outlined, and the types of microfluidic platforms commonly used in pediatric applications are introduced. Then, the specific uses of microfluidics in pediatrics are explored, with a focus on their roles in diagnostics, treatment, and research. Finally, the challenges currently faced in pediatric applications of microfluidics are discussed, and future prospects and potential advancements in pediatric healthcare are examined. By focusing on these key areas, this review aims to highlight the significance of microfluidic technology in pediatric medicine, providing researchers, clinicians, and technologists with valuable insights into the intersection of microfluidics and pediatric healthcare.

## 2 Microfluidics in Pediatrics

### 2.1 An Introduction to Microfluidics

Microfluidics, a multidisciplinary field at the intersection of engineering, physics, chemistry, and biology, refers to the manipulation and control of fluids at the sub‐millimeter scale, typically within channels tens to hundreds of micrometers wide [20, 31]. Microfluidic platforms are made from materials, such as PDMS, glass and PMMA, selected for properties including optical transparency and biocompatibility [32, 33, 34]. The underlying theory relies on fluid dynamics principles, particularly laminar flow regimes at low Reynolds numbers where fluid behavior remains predictable and stable [35]. To date, microfluidics has enabled transformative applications across biomedical research, chemistry, and environmental science [36, 37].

In biomedicine, it underpins lab‐on‐a‐chip diagnostics, organ‐on‐a‐chip drug screening and disease modeling, and droplet‐based single‐cell analysis [38, 39]. Beyond healthcare, it facilitates precise chemical synthesis and ultrasensitive environmental pollutant monitoring [40, 41]. Its technical advantages—minimal reagent consumption (critical for scarce pediatric samples), rapid response times (advantageous in pediatric acute care), and high‐throughput processing (enabling multiplexed analysis of trace volumes)—establish exceptional promise for pediatric applications [42].

### 2.2 Microfluidics in Pediatrics

The application of microfluidic technology in pediatric medicine can be categorized into four main types. The first is continuous microfluidics, which enables precise control of fluid movement within microchannels, allowing for efficient and high‐throughput sample analysis and processing. The second type, digital microfluidics, utilizes discrete droplets to perform sample manipulation, offering enhanced efficiency and precision for complex assays and tests. The third type, organ‐on‐a‐chip technology, replicates the microenvironments of human organs, creating advanced platforms for drug screening and disease modeling, which are particularly valuable for studying and treating pediatric diseases. Finally, paper‐based microfluidics leverages paper substrates to fabricate low‐cost and user‐friendly diagnostic devices, facilitating rapid and convenient testing and monitoring in both clinical and remote settings. These various microfluidic technologies, along with their applications in pediatrics, will be further explored in the following sections, as summarized in Table 1.

**TABLE 1:** A generalized summary of four microfluidic chip types used in pediatrics.

Type	Driving principles	Key features	Pediatric applications
Continuous	Pressure	Precise flow/reaction control, reduced reagent consumption, automation, clogging risk, complex design	Rapid diagnostics, real‐time biomarker monitoring and point‐of‐care testing
Digital	Electrostatic	Programmable droplet, low reagent consumption, on‐chip integration, electrode fouling, high cost	Infectious diseases and metabolic disorders in children
Organ‐on‐a‐chip	Pressure	Organ modeling, real‐time cellular monitoring, personalized medicine potential, high development costs, functional replication limitations	Developmental disorder studies, drug tests, and disease modeling
Paper‐based	Capillary action	Low cost, simple fabrication, user‐friendly limited reliability, environmental susceptibility	Portable diagnostic tests and affordable screening tools

#### 2.2.1 Continuous Microfluidics

Continuous microfluidics is a sophisticated technique that facilitates the manipulation of fluid streams within microscale channels (typically tens to hundreds of micrometers) [43]. Its core operation is fundamentally driven by pressure‐induced flow (e.g., via syringe pumps or pressure controllers), leveraging the dominant laminar flow regime inherent at such small scales. In laminar flow, fluids flow in parallel streams with minimal mixing via turbulence; mixing and reaction primarily occur through controlled molecular diffusion at the fluid interfaces. This precise control over fluid dynamics enables high‐resolution manipulation for mixing, reaction, and separation processes [44, 45]. The primary advantage of continuous microfluidics lies in its ability to provide real‐time dynamic control over fluidic processes, resulting in consistent and reproducible results [46]. This approach minimizes sample loss and contamination, as well as reduces reagent consumption, which is particularly beneficial in high‐throughput applications. Additionally, the integration of sensors and actuators within the microfluidic platform allows for the seamless monitoring and adjustment of experimental conditions, enhancing the accuracy and reliability of assays. Despite its numerous benefits, continuous microfluidics also presents certain limitations. The complexity of device fabrication and the need for precise alignment of microchannels can result in increased costs and time for development. Furthermore, the handling of highly viscous fluids or samples with particulates may pose challenges as clogging or flow disturbances can occur. Additionally, scaling up from microfluidic prototypes to larger, clinically applicable systems remains a significant hurdle. These challenges necessitate ongoing advancements in microfluidic technology to broaden its applicability and efficiency.

In pediatrics, continuous microfluidics has shown remarkable potential for improving diagnostic and therapeutic approaches. One prominent application is in the development of point‐of‐care diagnostic devices that can rapidly analyze the very small volumes of biological samples (e.g., blood drops, microliters of urine) that are often the only feasible or ethically acceptable option to obtain from neonates and young pediatric patients. These devices offer enhanced sensitivity and specificity for detecting various biomarkers and pathogens, which is crucial for early diagnosis and treatment of pediatric diseases. For example, microfluidic platforms have been employed for the detection of congenital disorders, infectious diseases, and metabolic conditions, providing timely and accurate results with minimal sample requirements [47]. This minimal invasiveness is a critical advantage in pediatric care. Additionally, continuous microfluidics facilitates the study of complex biological processes relevant to pediatric health, such as drug metabolism and developmental biology. By using microfluidic models to simulate the physiological environment of pediatric patients, it is possible to study how drugs or treatments affect growth and development at the microscopic scale. This approach enables the optimization of therapeutic strategies tailored to the unique physiological characteristics of children, ultimately leading to improved clinical outcomes.

#### 2.2.2 Digital Microfluidics

Digital microfluidics is an innovative technology that manipulates discrete droplets of fluids on a planar surface through the use of electric fields. This technology leverages the principle of electrostatic force to control the movement, mixing, and merging of droplets, enabling precise and programmable fluid handling [48, 49]. Compared to traditional continuous microfluidics, digital microfluidics provides greater flexibility and scalability in handling small volumes of liquid [50]. Its ability to operate with minimal reagent consumption and to integrate complex processes on a single platform makes it highly suitable for various applications including biochemical assays and point‐of‐care diagnostics. Despite its advantages, digital microfluidics also has certain limitations. One major drawback is its dependency on specialized materials and fabrication techniques, which can lead to higher initial costs and complexity in device production. In addition, the performance of digital microfluidics systems can be sensitive to environmental factors such as humidity and temperature, potentially affecting the reproducibility and reliability. Furthermore, while digital microfluidics excels in handling discrete droplets, it may be less effective in continuous flow processes than traditional microfluidic systems.

In the field of pediatrics, digital microfluidics has emerged as a powerful tool for diagnosis and treatment. Digital microfluidics is able to handle small sample volumes with high precision and is particularly beneficial for pediatric applications, where obtaining sufficient sample size can be challenging. Digital microfluidics systems are being utilized for developing advanced diagnostic assays that can detect diseases at early stages with minimal invasiveness. For instance, digital microfluidics‐based platforms are employed to perform multiplexed assays for screening metabolic disorders and infectious diseases in newborns, facilitating timely and accurate diagnosis [51, 52, 53]. Moreover, digital microfluidics supports personalized medicine by enabling the analysis of patient‐specific biomarkers from minute samples, which is crucial for tailoring treatments in pediatric patients.

#### 2.2.3 Organ‐on‐a‐Chip

Organ‐on‐a‐chip represents a groundbreaking advancement in biomedical engineering that aims to replicate the physiological functions of human organs on a micro‐scale [54, 55, 56]. Similar to continuous microfluidics, it utilizes precisely controlled pumps to generate pressure gradients, driving fluids containing nutrients, oxygen, and signaling molecules through intricate microchannels. By integrating living cells into microfluidic devices, these chips provide a platform for modeling organ‐specific environments and functions with high precision [57]. This continuous perfusion is crucial as it mimics the shear stress and dynamic biochemical milieu experienced by cells in vivo, surpassing the static conditions of traditional cell cultures. The primary advantages of organ‐on‐a‐chip include their ability to mimic the complex physiological interactions within human organs more accurately than traditional cell cultures or animal models [58]. This enhanced fidelity allows researchers to gain deeper insights into organ‐specific disease mechanisms and drug responses. Additionally, organ‐on‐a‐chip facilitates high‐throughput screening, reduces reliance on animal testing, and offers a more cost‐effective approach to studying human biology.

In pediatric research, organ‐on‐a‐chip has shown significant promise in advancing our understanding of developmental diseases and unique conditions in children. The precise control afforded by continuous microfluidics is particularly advantageous for modeling pediatric physiology. For instance, pediatric‐specific organ‐on‐a‐chip models can replicate the unique developmental stages of organs such as the liver, lungs, and kidneys, which are crucial for studying congenital disorders and age‐related pathologies [59]. Critically, microfluidics allows researchers to tailor flow rates and shear stresses to match the developing vasculature and lower blood flow velocities characteristic of infants and children, which cannot be readily achieved with conventional models. These models enable researchers to investigate how diseases manifest differently in children compared to adults, and to test the efficacy and safety of new treatments in a context that closely mimics pediatric physiology. Furthermore, the ability to sustain long‐term cultures under controlled flow conditions is essential for studying the prolonged processes of organ development and maturation, a key aspect of pediatric health and disease. Organ‐on‐a‐chip can also be used to study the impact of environmental factors and drug interactions on developing organs, providing valuable insights for tailoring pediatric therapies and improving clinical outcomes for younger patients [60].

#### 2.2.4 Paper‐Based Microfluidics

Paper‐based microfluidics is widely used in the biomedical field due to its inherent simplicity, cost‐effectiveness, and ease of use. The core driving principle relies on capillary action within the porous cellulose matrix of the paper substrate. This passive pumping mechanism spontaneously wicks and transports fluids along predefined hydrophilic channels without requiring external pumps or power sources. Utilizing absorbent paper as the substrate, this technology enables the development of low‐cost disposable devices capable of performing complex fluid manipulations without the need for external power sources [61, 62]. The primary advantages of paper‐based microfluidics include its affordability, which makes it accessible for resource‐limited settings, and its versatility in fabricating devices through simple processes such as wax printing or laser cutting. Additionally, the lightweight and portable nature of these devices enhances their applicability in point‐of‐care testing. However, there are notable limitations associated with paper‐based microfluidics. The performance of these devices can be affected by environmental factors such as humidity and temperature, which may influence the consistency and reliability of fluid flow and reaction outcomes. In addition, while paper‐based devices are ideal for rapid diagnostic applications, their limited capacity for integration with sophisticated detection systems can restrict their functionality in more complex assays [63].

In pediatrics, paper‐based microfluidics has demonstrated considerable promise for improving diagnostic and monitoring capabilities, offering several distinct advantages tailored to the needs of children and pediatric care settings. The inherent safety profile is paramount: the absence of sharp components, electricity, or complex moving parts minimizes the risks of injury during use, especially in uncooperative or young patients. The soft and flexible nature of paper is also more comfortable and less intimidating than rigid plastic or metal devices. Furthermore, the simplicity of operation—often requiring only a small sample (e.g., a drop of blood, urine, or saliva) applied to the device—makes it highly suitable for use by parents or caregivers at home, in community clinics, or in low‐resource settings with minimal training. For instance, these devices have been employed to develop innovative and user‐friendly diagnostic tools for detecting common pediatric conditions such as infections, anemia, and metabolic disorders [64]. The affordability and ease of use of paper‐based microfluidics make them particularly suitable for settings where access to advanced medical facilities is limited [65]. One notable application is the development of paper‐based tests for rapid detection of pathogens in bodily fluids, which is crucial for early diagnosis and management of infectious diseases in children. Moreover, paper‐based microfluidic platforms have been adapted for monitoring glucose levels in diabetic pediatric patients, offering a simple yet effective way to track and manage their condition [66].

## 3 Application of Microfluidics in Pediatrics

This section will discuss the diverse applications of microfluidic technology in pediatric medicine, with a focus on its roles in diagnosis, research, and treatment.

### 3.1 Pediatric Disease Diagnosis

Rapid diagnosis of diseases is crucial for timely treatment, particularly in pediatrics, where several challenges complicate the diagnostic process. Due to the limited body weight and cooperation of children, the volume of diagnostic samples that can be obtained, such as blood, is often minimal [67]. Furthermore, children are highly sensitive to invasive procedures such as lumbar punctures and bone marrow biopsies. Current conventional diagnostic methods typically require large sample volumes and are time‐consuming, making them difficult to implement, especially in resource‐limited settings. Therefore, it is crucial to develop diagnostic methods that are efficient, reliable, and economically viable.

Microfluidic technology offers a promising solution to these challenges. It requires only a minimal amount of blood or bodily fluid, making it particularly suitable for pediatric sample collection. Additionally, microfluidic platforms can deliver diagnostic results within minutes or hours, significantly reducing the waiting time for children undergoing testing. Some microfluidic platforms can even be designed as non‐invasive devices, making them more child‐friendly and ideal for use in home‐based or primary healthcare settings.

#### 3.1.1 Pediatric Infectious Diseases Diagnosis

**FIGURE 2:**
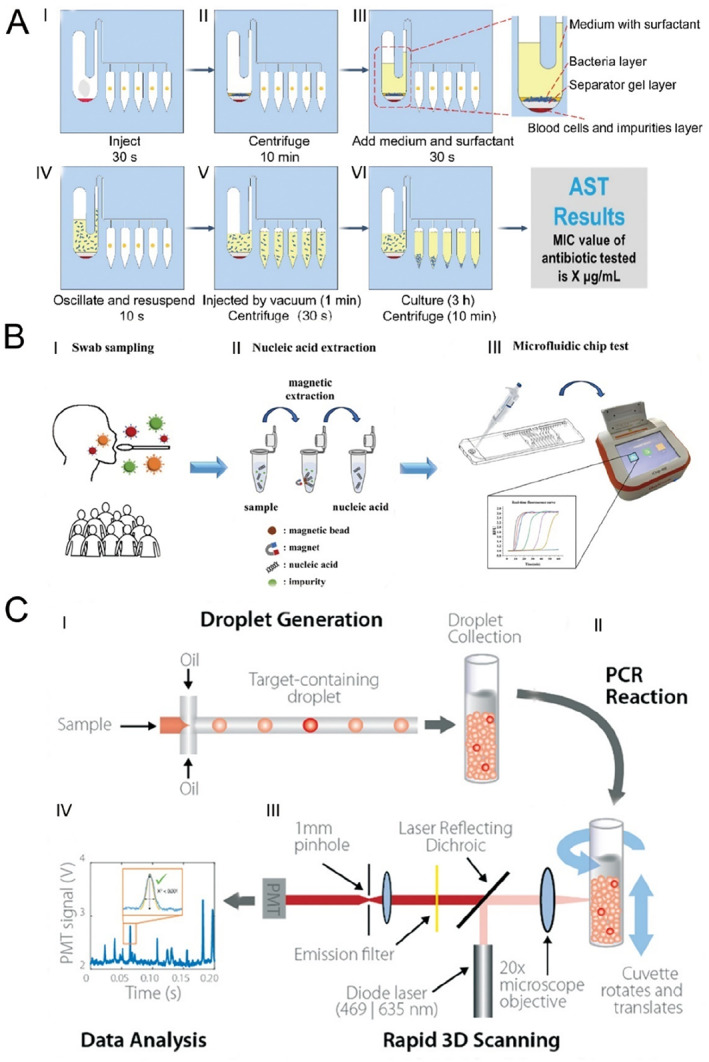
Microfluidic technology for the diagnosis of pediatric infectious diseases. (A) Workflow for rapid bacterial separation and antimicrobial susceptibility testing from positive blood cultures using the microfluidic chip. Reproduced with permission [47]. Copyright 2023, American Chemical Society. (B) Schematic illustration of the microfluidic LAMP‐based real‐time fluorescence assay for the detection of multiple respiratory pathogens. Reproduced with permission [72]. Copyright 2024, Royal Society of Chemistry. (C) Workflow for rapid bacterial identification and antimicrobial susceptibility testing using the microfluidic chip. Reproduced with permission [73]. Copyright 2020, Royal Society of Chemistry.

Infectious diseases are among the most common illnesses in pediatric populations, with respiratory and gastrointestinal infections being the primary forms [68, 69]. Rapid identification of the causative pathogens is critical for effective diagnosis and treatment, as delays may lead to disease progression or deterioration. Although pathogen cultures, including bacteria, viruses, and fungi, are regarded as the “gold standard” for diagnostic confirmation, this approach is time‐consuming and often exhibits reduced sensitivity, particularly for viruses and bacteria that are difficult to culture [70]. In contrast, microfluidic technology has significantly accelerated the diagnostic process while maintaining accuracy comparable to traditional methods [71]. For instance, Zhu et al. [47] developed a microfluidic chip capable of rapidly extracting bacteria from positive blood cultures within 10 min and providing antimicrobial susceptibility information within 3 h (Figure 2A). The chip, which includes a bacteria separation chamber, multiple sensitivity analysis testing chambers, and connecting channels, utilizes centrifugal enrichment technology to detect different antibiotic‐induced phenotypic growth within hours in the sensitivity testing chambers. Successful application of this chip in diagnosing clinically positive blood cultures demonstrated a classification consistency of 93.3% with standard methods, underscoring its significant potential for clinical diagnosis.

In addition to improving the efficiency and accuracy of blood culture diagnostics, microfluidic technology has also enhanced other diagnostic approaches for infectious diseases. For example, Liu et al. [72] introduced a microfluidic chip designed for the rapid identification of various respiratory pathogens. This device employs a ten‐channel microfluidic architecture integrated with LAMP technology and real‐time fluorescence monitoring (Figure 2B). By providing a sealed reaction environment, the chip minimizes aerosol contamination risks and improves detection precision. Additionally, the associated detection system enables real‐time automatic fluorescence data acquisition and visualization, simplifying the result interpretation. The approach successfully identified nucleic acids from H1N1 influenza virus, mycoplasma pneumoniae, respiratory syncytial virus type A, and SARS‐CoV‐2, achieving detection thresholds as low as 10^3^–10^4^ copies/mL. Clinical evaluations demonstrated high diagnostic sensitivity (92.00%) and perfect specificity (100.00%), with the capability to differentiate mixed infection samples.

Similarly, Abram et al. [73] developed a novel rapid diagnostic platform that integrates droplet‐based microfluidics with PCR testing for the detection of bacterial pathogens (Figure 2C). This system facilitates the direct detection of bacteria and assessment of antibiotic susceptibility using whole blood samples, bypassing the need for culturing or complex sample preparation. In blinded evaluations with clinical isolates, it achieved perfect sensitivity and specificity in identifying pathogens harboring particular resistant genes. Additionally, it successfully recognized a wide variety of antibiotic resistance determinants and bacterial species, encompassing both Gram‐positive and Gram‐negative bacteria, without the reliance on blood culture.

#### 3.1.2 Genetic and Metabolic Diseases Screening

Genetic and metabolic diseases represent a significant subset of pediatric diseases. These conditions are often diagnosed during the neonatal period or even during prenatal screening. In China, newborns undergo routine heel‐prick blood sampling to screen for four common inherited metabolic disorders: glucose‐6‐phosphate dehydrogenase (G6PD) deficiency, congenital adrenal hyperplasia, phenylketonuria (PKU), and congenital hypothyroidism [74]. Early diagnosis is crucial for timely intervention and treatment, helping to prevent complications such as developmental delays, intellectual disabilities, and even mortality due to damage to vital organs such as the brain, liver, and kidneys [75]. Currently, common diagnostic methods for inherited metabolic disorders include tandem mass spectrometry, enzyme activity assays, and genetic testing. Microfluidic technologies, with their unique advantages, can be integrated with these diagnostic techniques to provide faster, more accurate, and cost‐effective diagnosis.

**FIGURE 3:**
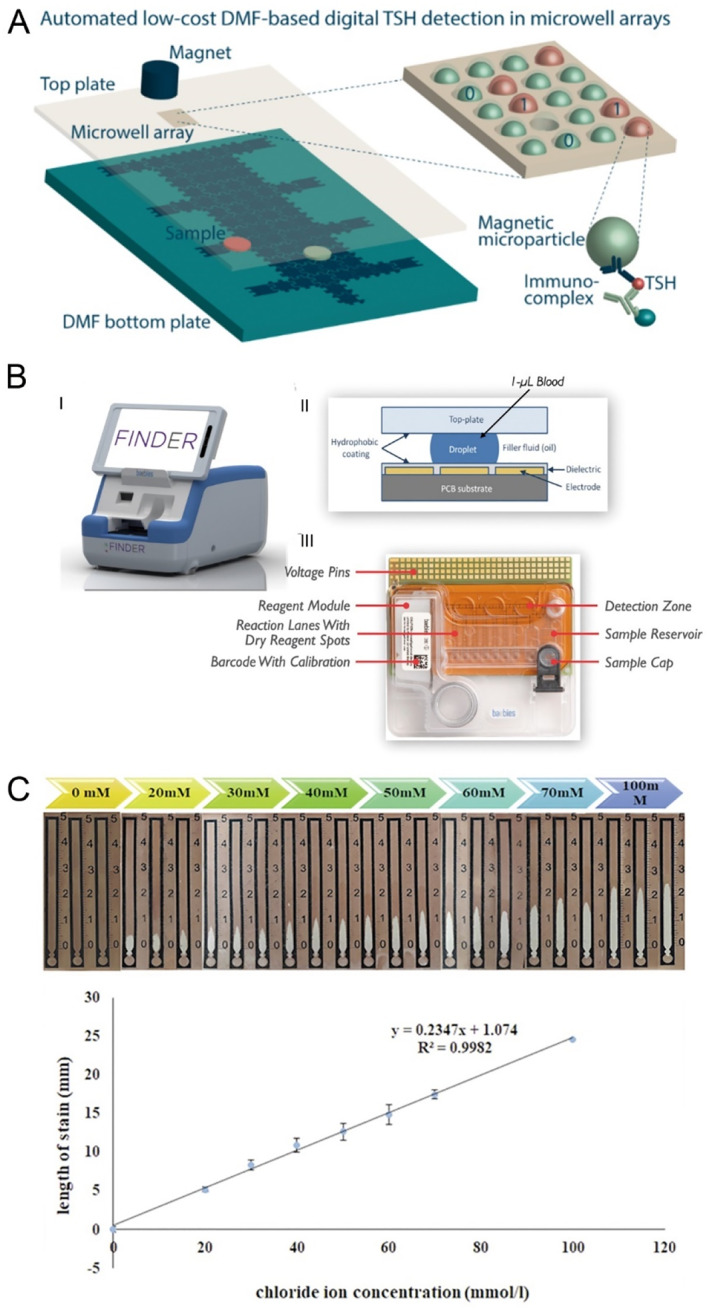
Microfluidic technology for the screening for pediatric genetic and metabolic diseases. (A) Schematic diagram of TSH concentration detection based on digital microfluidic technology. Reproduced with permission [53]. Copyright 2022, American Chemical Society. (B) Photograph of the digital microfluidic device for G6PD concentration detection, a schematic of the underlying principle of digital microfluidic technology, and a detailed illustration of the device chip. Reproduced with permission [52]. Copyright 2021, Elsevier. (C) Device images at different chloride ion concentrations and Calibration curve of chloride ion concentration versus white stain length. Reproduced with permission [64]. Copyright 2019, Elsevier.

Congenital hypothyroidism (CH) is an endocrine disorder characterized by insufficient thyroid function, typically diagnosed at birth or shortly thereafter [76]. This condition is primarily marked by low levels of thyroid hormones, particularly thyroxine, which can lead to significant issues in growth and cognitive development. Leirs et al. [53] devised a novel microfluidic detection platform that was developed by leveraging the ability of digital microfluidics to automate operations and analyze extremely small sample volumes (Figure 3A). This was combined with low‐cost disposable chips featuring micropore arrays fabricated via roll‐to‐roll processes. By optimizing reagent concentrations and incubation times, the platform was employed for thyroid‐stimulating hormone (TSH) detection. In experiments, using only 1.1 μL of sample, the seeding efficiency of the hydrophobic micropores was 97.6% ± 0.6%, with a calculated detection limit of 0.0013 μIU/mL, demonstrating the platform's significant potential.

G6PD deficiency is a hereditary enzyme deficiency primarily affecting red blood cells. In newborns, G6PD deficiency typically manifests within the first few days after birth, potentially leading to jaundice and hemolytic anemia. Due to the immature hepatic function in neonates, there is an increased risk of hyperbilirubinemia. Therefore, early screening and monitoring are crucial for the timely identification and management of potential complications. A research team in the United States utilized a digital microfluidic platform employing electrowetting to continuously mix sample droplets with lysis buffer droplets until a dilution of 1/27 was achieved (Figure 3B) [52]. The G6PD concentration was then determined by measuring the conversion of NADP to NADPH. When comparing this digital microfluidic method with two traditional measurement techniques, they found minimal variability and bias between the results, demonstrating the effectiveness of this method in screening for G6PD deficiency.

Cystic fibrosis (CF) is an inherited disorder of the exocrine glands that predominantly impacts the respiratory and gastrointestinal systems. It is marked by persistent obstructive lung disease, insufficiency of pancreatic exocrine function, and heightened concentrations of electrolytes in sweat [77]. Sweat chloride testing is commonly utilized for newborn screening of CF. However, existing methods for measuring chloride levels in sweat face limitations related to low selectivity and the need for relatively large sample volumes [78]. To address these issues, Maryam Taghizadeh‐Behbahani and colleagues [64] developed a paper‐based microfluidic device (Figure 3C). Upon the addition of chloride‐containing sweat samples, the sweat traverses through channels, resulting in the formation of AgCl precipitates, which manifest as white spots. The length of these spots in the channel correlates directly with the chloride ion concentration in the sweat. This process requires only 2.0 μL of sample solution and completes analysis within 1 min while maintaining low costs. Additionally, the device exhibits a broad measurement range, encompassing both healthy and at‐risk levels, with minimal deviation compared to standard analytical methods.

#### 3.1.3 Hematologic Malignancies Diagnostics and Prognosis

Hematologic cancers in children, including leukemia and lymphoma, represent some of the most frequently diagnosed malignancies in pediatric populations [79]. These disorders are marked by the uncontrolled growth of blood cells, which disrupts normal bone marrow activity and results in anemia as well as compromised immune functionality. Early and accurate diagnosis is critical for determining appropriate treatment strategies, and prognosis is closely tied to timely intervention and disease monitoring. In clinical practice, the diagnosis and prognosis of pediatric hematologic malignancies often rely on a variety of genetic and molecular assays. High‐throughput microfluidic technologies have introduced significant advancements in this domain by enabling the efficient analysis of exceedingly small sample volumes, thereby enhancing detection capabilities beyond those of traditional methodologies. Microfluidic platforms facilitate simultaneous multiplexed assays, allowing for comprehensive genetic and molecular profiling with greater speed and accuracy. Additionally, the minimally invasive nature of microfluidic techniques is particularly advantageous in pediatric settings, as it reduces patient discomfort and the risk associated with sample collection. This low invasiveness not only improves patient compliance but also minimizes the psychological and physical burden on young patients. Moreover, the integration of microfluidic systems can lead to substantial cost reductions in diagnostic testing through streamlined workflows and reduced reagent consumption.

**FIGURE 4:**
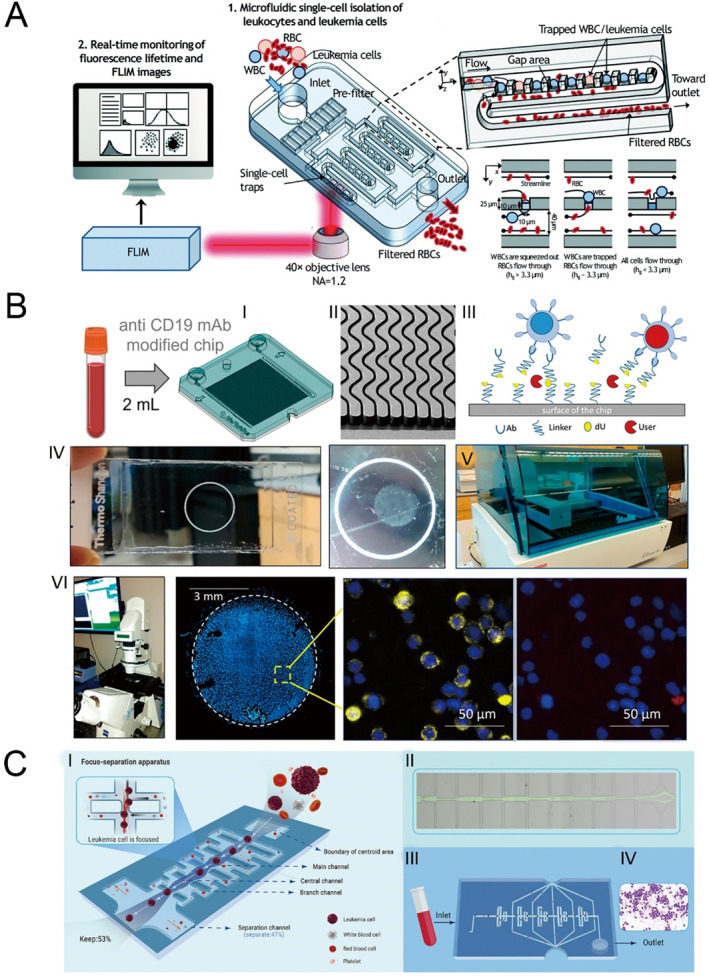
Microfluidic technology for the pediatric hematologic malignancies diagnostics and prognosis. (A) Schematic diagram and flow of a microfluidic platform for label free separation and rapid identification of single blood leukemia cells based on fluorescence lifetime imaging microscopy. Reproduced with permission [80]. Copyright 2018, Royal Society of Chemistry. (B) Workflow for processing patient blood samples to search for CLCs. Reproduced under terms of the CC‐BY license [49]. Copyright 2024, The Authors, published by MDPI. (C) Principle and methodology of utilizing CTC enrichment chips in MRD detection. Reproduced with permission [83]. Copyright 2023, The Authors, published by Elsevier.

Efficient screening and isolating individual leukemic cells from blood samples is essential for the early diagnosis of leukemia. Nevertheless, the considerable similarity in size between leukemic cells and the more prevalent white blood cells (WBCs) poses a significant challenge to their separation and identification in peripheral blood. Traditional approaches often rely on immunolabeling or cytogenetic methods for accurate detection. A microfluidic platform has been introduced for the rapid and label‐free detection of individual leukemic cells [80]. This system integrates a high‐throughput single‐cell capture array for size‐selective separation of red blood cells (RBCs) with phasor‐based fluorescence lifetime imaging microscopy (phasor‐FLIM) to differentiate leukemic cells (Figure 4A). By measuring the metabolic shift between free and bound nicotinamide adenine dinucleotide (NADH), it provides an indirect assessment of cellular metabolic states. The microfluidic array incorporates 1600 densely packed addressable single‐cell traps, allowing for concurrent RBC removal and efficient capture of white blood cells (WBCs) or leukemic cells. It is optimized for low‐magnification imaging and fast fluorescence‐based analysis. This approach showcases the ability to achieve high‐density, size‐dependent single‐cell isolation, RBC exclusion, and label‐free leukemic cell identification using non‐invasive metabolic imaging, underscoring its promise for early leukemia detection.

Minimal Residual Disease (MRD) serves as the most significant indicator of prognosis in individuals diagnosed with acute lymphoblastic leukemia. Defined as the detection of leukemic cells within the bloodstream or bone marrow, MRD plays a vital role in evaluating the effectiveness of therapeutic interventions. Researchers in the United States have introduced a microfluidics‐based MRD (MF‐MRD) detection technique, enabling regular assessment of circulating leukemia cells (CLCs) using blood samples [49]. The microfluidic chip utilizes anti‐CD19 antibodies, immobilized on its surface, to selectively isolate B‐lineage cells including CLCs. Following isolation, these cells can be retrieved for downstream analyses, such as immunophenotyping, fluorescence in situ hybridization (FISH), or molecular examination of CLC mRNA or gDNA (Figure 4B). This approach enables the detection of MRD using blood volumes as small as 2 mL, presenting a notable improvement over conventional multiparameter flow cytometry (MFC‐MRD) by effectively handling limited sample quantities.

Circulating tumor cells (CTCs) can be isolated from normal cells not only by their biological traits but also through distinct physical characteristics [81]. Techniques such as filtration, density gradient centrifugation, and inertial focusing are frequently employed, relying mainly on variations in cell size and flexibility. However, these approaches often struggle with low purity and efficiency [82]. Microfluidic devices, with their microscale dimensions, enable precise manipulation of particles and single‐cell analysis, making them increasingly attractive for CTC isolation. A research team has designed a microfluidic system that effectively isolates circulating tumor cells (CTCs) within a defined size range [83]. Distinct from other methods relying solely on physical characteristics, this approach integrates filtration with hydrodynamic focusing. The device incorporates multiple channel arrays, termed focusing structures, alongside two additional channels designated as separation structures (Figure 4C). Within the focusing sections, branch channels of varying lengths generate differential flow resistances, directing larger cells, such as CTCs, into the central channel, where they are continually guided toward the centerline by interactions with the sidewalls. After passing through several focusing structures, CTCs concentrate in the middle of the channel, while smaller cells remain at the edges. The separation channels, with specific flow resistances, then isolate the fluid devoid of target cells, effectively increasing the CTC concentration. In functional tests, the device demonstrated capture efficiencies greater than 90% across flow rates ranging from 6 to 40 mL/h. Furthermore, the removal rate of non‐target cells slightly outside the critical size range exceeded 90%. This chip is suitable for CTC detection in solid tumors, offering sensitivity comparable to that of flow cytometry (MFC) and quantitative PCR (qPCR), with an overall detection cost of less than 50 RMB, significantly lower than MFC or PCR.

#### 3.1.4 Paper‐Based Microfluidics for At‐Home Diagnostics or Resource‐Limited Settings

Paper‐based microfluidic devices utilize porous paper substrates to facilitate the movement and analysis of body fluids such as sweat and saliva, which are less invasive compared to blood sampling [84, 85]. Therefore, it represents a versatile approach to diagnostic testing, particularly beneficial for pediatric care in home settings. Compared to traditional blood draws, which are challenging and distressing, Paper‐based microfluidic systems offer the capability to monitor various physiological parameters at home, enabling parents and caregivers to track health indicators such as glucose levels and electrolyte concentrations without frequent clinic visits [86, 87]. In addition to their utility in home‐based monitoring, paper‐based microfluidic devices are also valuable in regions with limited medical resources. Their affordability and ease of use enable widespread deployment in low‐income areas where advanced diagnostic infrastructure may be lacking. Examples include portable diagnostic tests for infectious diseases, such as malaria or tuberculosis, which can be performed using paper‐based assays to quickly and effectively identify the presence of pathogens with minimal equipment [88, 89].

**FIGURE 5:**
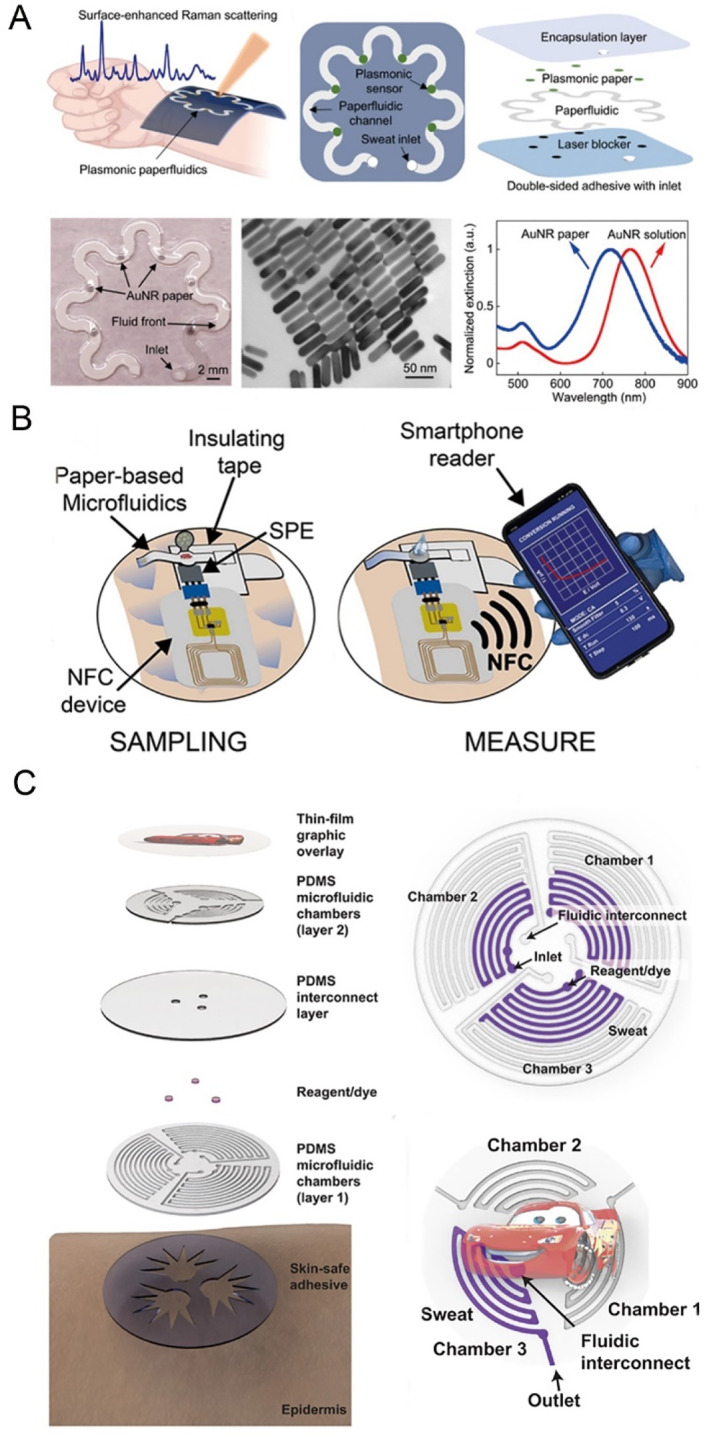
Microfluidic technology for at‐home diagnostics or resource‐limited settings. (A) Conceptual illustration, schematics, and characterization of a wearable plasmonic paperfluidic device for real‐time quantitative analysis of sweat loss, rate, and metabolites. Reproduced under terms of the CC‐BY license [65]. Copyright 2022, The Authors, published by American Association for the Advancement of Science. (B) Illustration of cortisol monitoring during physical activity using paper‐based microfluidics. Reproduced with permission [90]. Copyright 2023, Elsevier. (C) Exploded view of the microfluidic device called “sweat sticker” and the geometry of the microfluidic channels. Reproduced with permission [91]. Copyright 2021, The Authors, published by American Association for the Advancement of Science.

Mogera et al. [65] developed a microfluidic system integrated with wearable plasmonic paper, enabling real‐time simultaneous quantification of sweat loss, secretion rate, and metabolic components in perspiration (Figure 5A). Utilizing label‐free surface‐enhanced Raman spectroscopy (SERS) as its core sensing mechanism, the system delivers molecular “fingerprint” data essential for identifying various analytes. This device has demonstrated its effectiveness in detecting uric acid levels in sweat, covering ranges pertinent to both normal physiological conditions and pathological states.

Another research team from Italy reported a paper‐based microfluidic device to detect the cortisol in sweat [90]. They used wax printing and laser‐cutter technique to make the paper‐based microfluidic pattern and used magnetic beads functionalized with monoclonal antibodies to recognize the cortisol in sweat. The reliability of this sustainable paper‐based device was well demonstrated by the Volunteer experiment (Figure 5B). The determination of chloride concentration in sweat can aid in diagnosing cystic fibrosis, a disease commonly found in children.

Ray et al. [91] developed a flexible epidermal microfluidic system, named the “sweat sticker,” to facilitate efficient and rapid sweat collection and analysis (Figure 5C). This innovative device adheres closely to the skin, ensuring minimal sweat loss. Furthermore, it enables real‐time measurement of chloride levels in sweat through smartphone‐based image analysis, providing precision comparable to conventional techniques.

**FIGURE 6:**
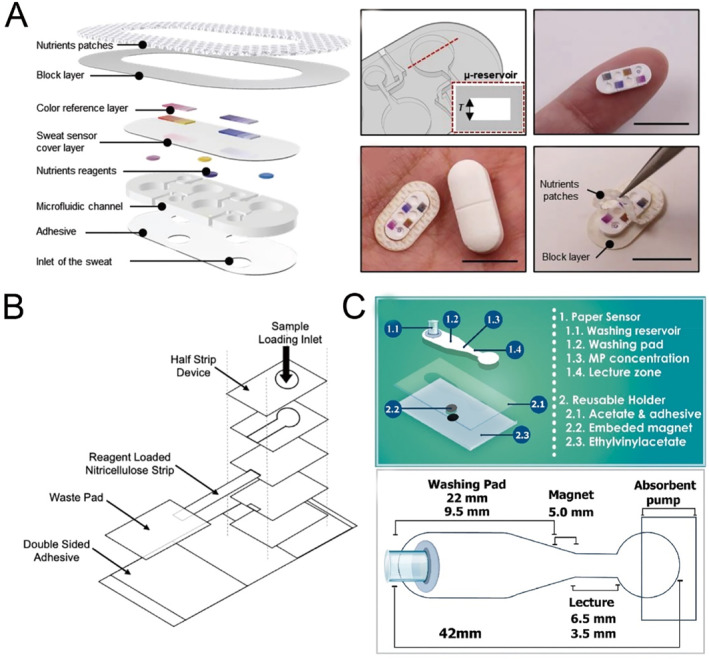
Microfluidic technology for at‐home diagnostics or resource‐limited settings. (A) Schematic illustrations and optical images of a miniaturized sweat microfluidic device designed to measure four key sweat nutrients via colorimetric analysis. Reproduced under terms of the CC‐BY license [92]. Copyright 2022, The Authors, published by Wiley‐VCH GmbH. (B) Schematic illustration of the microfluidic device assembly for iron deficiency diagnosis. Reproduced with permission [94]. Copyright 2024, Royal Society of Chemistry. (C) Schematic representation of the detection system and dimensions of the single‐piece paper device. Reproduced with permission [95]. Copyright 2022, Elsevier.

Kim et al. [92] have designed a microfluidic system that easily and non‐invasively captures human sweat without requiring any supporting electronic devices (Figure 6A). This system allows for the simple and rapid colorimetric evaluation of multiple essential nutrients in sweat including vitamin C, calcium, zinc, and iron. Additionally, the microfluidic system integrates a transdermal patch, which passively and continuously delivers these substances to the body during wear. The researchers also validated the correlation between the temporal dynamics of these nutrient concentrations in sweat and their corresponding concentrations in blood. Furthermore, they investigated the impact of food and beverage consumption on these concentrations, providing valuable insights for personalized nutrition.

The integration of paper‐based microfluidics into pediatric diagnostics enhances accessibility and affordability, empowering caregivers and healthcare workers with essential tools for timely and accurate health assessments. Iron deficiency anemia (IDA) is common in infants and young children and existing diagnostic methods rely on invasive analysis of stored iron in ferritin [93]. Prakobdi et al. [94] have designed a microfluidic device that detects iron content in children's saliva (Figure 6B). They integrated a capillary‐driven microfluidic device into a lateral flow system utilizing a nitrocellulose membrane, which exhibited a linear response to the collected saliva samples, demonstrating its potential application in remote and resource‐limited settings.

A paper‐based microfluidic platform has been created for the highly specific and sensitive identification of Plasmodium falciparum lactate dehydrogenase (Pf‐LDH), a key biomarker for malaria (Figure 6C) [95]. This system integrates a single‐step magneto‐immunoassay with a microfluidic paper substrate and a portable fluorescence reader. The assay procedure includes a brief 5‐min incubation of magnetic particles coated with antibodies, a biotinylated detection antibody, and an enzymatic signal enhancer, followed by fluorescence measurement using an affordable handheld device. Quantitative Pf‐LDH detection was achieved in under 20 min, with a sensitivity threshold of 0.92 ng/mL, corresponding to 4.6 parasites/μL. The performance is comparable to ELISA and surpasses that of commercial rapid diagnostic tests (RDTs), highlighting the promise of fluorescent paper‐based microfluidics for use in resource‐limited settings.

### 3.2 Pediatric Disease Research

Pediatric disease research is critical for understanding developmental pathophysiology and advancing tailored therapies for children, a population with distinct physiological and metabolic profiles compared to adults. However, such research faces significant hurdles: ethical constraints limit patient recruitment and tissue sampling, in vivo models often fail to recapitulate human developmental processes, and conventional in vitro systems lack the complexity to model dynamic organ interactions. These limitations impede disease mechanism discovery and delay drug development in pediatric populations.

Microfluidic technologies offer transformative solutions to these challenges. Organ‐on‐a‐chip platforms, incorporating patient‐derived cells and biomimetic microenvironments, enable the development of pediatric disease models that mirror tissue‐tissue interfaces and mechanical cues. Furthermore, microfluidics facilitates high‐throughput drug screening by allowing parallelization of miniature tissue cultures with precise perfusion control, drastically accelerating compound testing. Integrated sensors and automated fluid handling further empower scalable multi‐parametric analysis—from real‐time cytokine secretion monitoring to single‐cell transcriptomics—uncovering molecular signatures inaccessible via bulk methods. Collectively, these capabilities position microfluidics as a pivotal tool for overcoming the scale and complexity barriers in pediatric research.

#### 3.2.1 Pediatric Disease Modeling Based on Organ‐on‐a‐Chip

Disease related model holds significant importance in the field of pediatrics research. Developing and analyzing disease models enables pediatricians and researchers to better understand disease mechanisms and progression, identify promising therapeutic targets, evaluate drug efficacy and safety, and improve diagnostic approaches [96, 97]. Currently, common disease models include cell models, which primarily use cultured cells or cell lines to investigate molecular and cellular mechanisms, and animal models, which involve using animals (e.g., mice, rats, zebrafish) to simulate human diseases and study their systemic effects.

While both animal and cell models have played significant roles in disease research, they possess limitations. Notably, substantial physiological and genetic differences exist between animals and humans, and maintaining animal models can be costly [98, 99]. Conversely, cell models are limited to individual cells and lack the complex interactions found within tissues and organs in vivo. Additionally, the long‐term maintenance and stability of cell models present challenges. Organ‐on‐a‐Chip is an innovative platform that combines microfluidic technology and cell culture to simulate the microenvironment and functions of human organs [58, 100]. It offers a more physiologically relevant research platform compared to traditional cell models and animal models by more accurately mimicking the microenvironment, structure, and function of human organs [101, 102]. Organ‐on‐a‐Chip technology allows for the simultaneous conduct of multiple experiments, making it suitable for high‐throughput drug screening and toxicity testing, thereby accelerating the drug development process [103]. Additionally, it can utilize patient‐derived cells to construct personalized disease models, aiding in the development of targeted therapies for specific patients. Moreover, Organ‐on‐a‐Chip technology can integrate multiple organ chips to study the interactions between different organs and the mechanisms of systemic diseases, providing a more comprehensive disease model [104]. The organ‐on‐a‐chip technology, aided using microfluidic techniques, enables the simulation of organ functions and facilitates the simultaneous modeling of multiple diseases. This advancement offers the potential for more personalized and precise simulations of pediatric conditions, thereby enhancing the accuracy and efficiency of treatment approaches.

**FIGURE 7:**
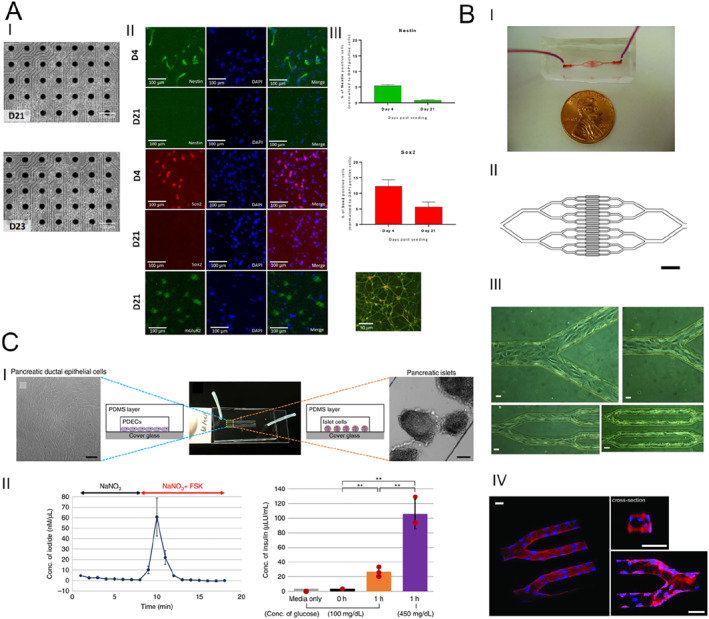
Organ‐on‐a‐chip models for pediatric disease. (A) Cell characterization of glutamatergic neurons and high‐grade pediatric glioma lines. Reproduced with permission [59]. Copyright 2021, *MyJoVE Corporation*. (B) An in vitro microfluidic model simulating the microvasculature, designed to study disease processes involving biophysical interactions between cells. Reproduced with permission [105]. Copyright 2011, American Society for Clinical Investigation. (C) A single‐channel microfluidic chip was designed to replicate the structure of a pancreatic duct, featuring branching pathways and progressively narrowing diameters. Reproduced under terms of the CC‐BY license [106]. Copyright 2019, The Authors, published by Springer Nature.

Pediatric high‐grade gliomas (PHGG) are associated with a dismal prognosis in children and adolescents, underscoring the urgent need for innovative treatment strategies. To address this, researchers have established a functional in vitro model by co‐culturing human induced pluripotent stem cell (hiPSC)‐derived glutamatergic neurons with PHGG cells while simultaneously monitoring their electrophysiological activity (Figure 7A) [59]. Findings reveal a marked enhancement in neuronal excitability when tumor cells are present. This platform offers valuable insights and could pave the way for the development of novel therapeutic strategies targeting PHGG.

A research group from the United States has introduced an in vitro microfluidic model of the microvasculature aimed at investigating blood disorders such as sickle cell disease (SCD) and hemolytic‐uremic syndrome (HUS) (Figure 7B) [105]. These disorders are characterized by pathological biophysical interactions involving blood cells, endothelial cells, and soluble mediators, which contribute to thrombus development and microvascular blockage. This model can simulate the biophysical changes associated with these diseases under controlled flow conditions and can also be utilized to investigate the effects of drugs on these conditions, including the role of hydroxyurea in SCD microvascular occlusion and the effect of eculizumab in hemolytic‐uremic syndrome. The versatility and clinical relevance of this microsystem have been demonstrated, making it suitable for studying the pathophysiology of blood disorders and drug discovery.

Cystic fibrosis (CF) is a genetic disorder stemming from dysfunction in the CF Transmembrane Conductance Regulator (CFTR). Pancreatic islets, responsible for insulin production, are situated near the pancreatic ducts, suggesting that disrupted cell‐cell communication between pancreatic ductal epithelial cells (PDECs) and islet cells may contribute to the disease. To investigate this hypothesis, a novel pancreas‐on‐a‐chip was developed (Figure 7C) [106]. The results reveal that defective CFTR function in PDECs markedly hinders insulin secretion by islet cells. This innovative platform provides a valuable tool for studying CF‐related pathophysiology, offering new insights into PDEC‐islet interactions, supporting the assessment of potential therapeutic approaches and enhancing the understanding of pancreatic function.

#### 3.2.2 Drug Screening for Pediatric Diseases

Drug screening is a critical component of pediatric disease research, facilitating the identification of effective and safe therapeutic agents tailored specifically for young patients. Pediatric drug development presents unique challenges due to the physiological differences between children and adults, including variations in drug metabolism, pharmacokinetics, and potential side effects [107]. Consequently, robust drug screening processes are essential to ensure that treatments are both effective and appropriate for the pediatric population. Advances in drug screening methodologies are crucial for discovering new therapies and optimizing existing treatments for a wide range of pediatric conditions.

Microfluidic technology has significantly advanced the field of drug screening by enhancing precision, efficiency, and scalability. These platforms can handle minute volumes of biological samples and reagents, allowing for high‐throughput screening of compounds with reduced reagent consumption and faster turnaround times [54]. Furthermore, microfluidic systems enable the integration of complex biological assays, including cell‐based and tissue‐based models, within a compact and automated system [108].

**FIGURE 8:**
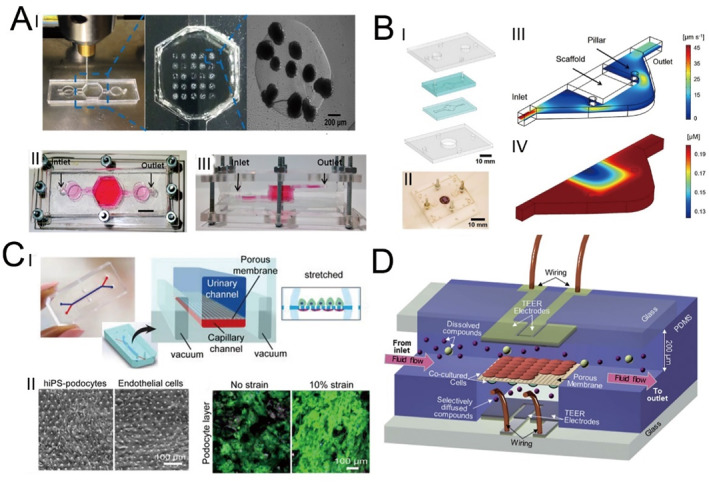
Microfluidic technology for drug screening in pediatric diseases. (A) Schematic and photograph of a liver‐on‐a‐chip structure for drug screening. Reproduced with permission [109]. Copyright 2016, IOP Publishing. (B) Schematic and photograph of a heart‐on‐a‐chip structure for drug screening. Reproduced with permission [110]. Copyright 2016, Elsevier. (C) Schematic and photograph of a glomerulus‐on‐a‐chip structure for drug screening. Reproduced with permission [111]. Copyright 2017, Springer Nature. (D) Schematic diagram of the microfluidic in vitro model of the blood–brain barrier for drug screening. Reproduced with permission [112]. Copyright 2012, Royal Society of Chemistry.

The liver is the primary organ for assessing drug toxicity. Researchers utilize HepG2/C3A cells to fabricate three‐dimensional liver organoids via bioprinting methods, encapsulating them within photocrosslinkable gelatin methacryloyl (GelMA) hydrogels, thus constructing a liver‐on‐a‐chip platform [109]. This platform assesses the hepatotoxicity of acetaminophen, resulting in decreased cell density, metabolic activity, and the production of biomarkers, which mirrors findings from animal models and other in vitro systems (Figure 8A).

Yu Shrike Zhang and colleagues [110] employed 3D bioprinting technology to develop heart organoids incorporating cardiomyocytes (CMs) and endothelial cells derived from human induced pluripotent stem cells (hiPSCs) (Figure 8B). These organoids were designed to replicate endothelialized myocardial tissue. The cardiac toxicity of doxorubicin, a widely used chemotherapy drug, was assessed by monitoring the beating rate of CMs using optical microscopy.

Musah et al. [111] engineered a glomerulus‐on‐a‐chip by directing the differentiation of hiPSCs into mature podocytes, which were subsequently co‐cultured with human glomerular endothelial cells (Figure 8C). This platform successfully reproduced key glomerular functions, such as blood filtration and urinary clearance, and effectively modeled podocyte damage caused by Adriamycin, an anticancer agent.

Booth and collaborators [112] created a microfluidic blood–brain barrier (mBBB) chip by integrating PDMS layers, electrode components, and polycarbonate membranes, with astrocytes and endothelial cells seeded on opposite sides of the membrane (Figure 8D). This device was utilized to evaluate the transport kinetics of dextran at various molecular weights (4, 20, and 70 k) and propidium iodide, showcasing its potential for preclinical drug testing applications.

#### 3.2.3 High Throughput Research

As pediatric research continues to advance, similar to other fields of medical science, the methodologies and approaches are constantly evolving, with a growing emphasis on high‐throughput techniques. Currently, widely used approaches include high‐throughput sequencing, high‐throughput proteomics, and single‐cell sequencing, among others [113, 114]. The inherent high‐throughput capabilities of microfluidic technology make it particularly well‐suited to complement these methods, providing significant advantages for pediatric disease research [38, 115]. By enabling the efficient handling of small sample volumes, precise control of experimental conditions, and integration of multiple analytical steps, microfluidics enhances the potential for more rapid and accurate data acquisition, thereby accelerating the discovery of disease mechanisms and the development of targeted therapies in pediatrics.

**FIGURE 9:**
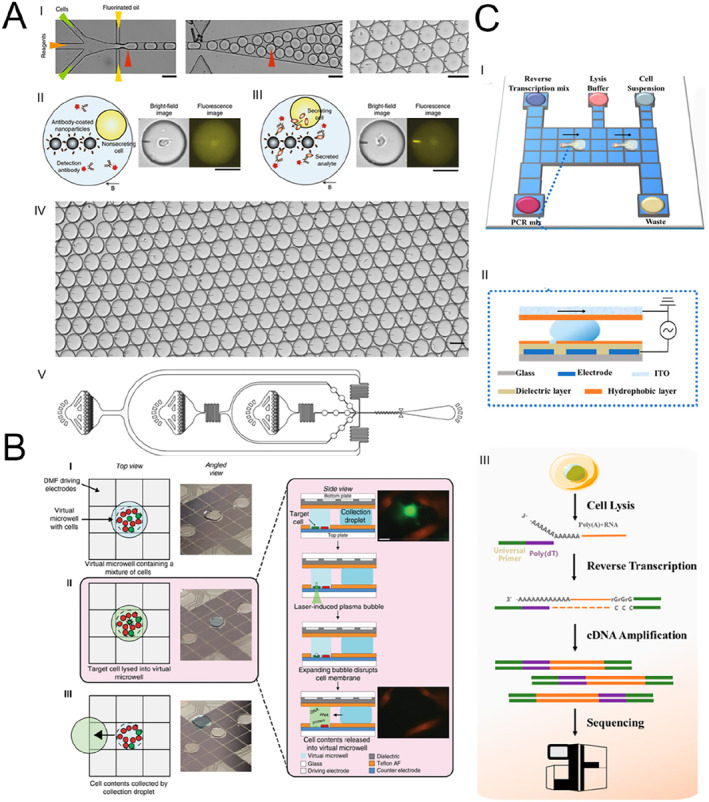
Microfluidic technology for pediatric high throughput research. (A) Workflow schematic of a single‐cell secretion assay within microfluidic droplets. Reproduced with permission [116]. Copyright 2020, The Authors, published by Springer Nature. (B) Workflow schematic of digital microfluidic isolation of single cells for omics analyses. Reproduced under terms of the CC‐BY license [117]. Copyright 2020, The Authors, published by Springer Nature. (C) Schematic illustration of digital RNA sequencing. Reproduced with permission [118]. Copyright 2020, American Chemical Society.

A research group has introduced DropMap, a microfluidic platform designed to simultaneously analyze secretion dynamics and other cellular characteristics from tens of thousands of individual immune cells (Figure 9A) [116]. This technology isolates single cells within 50‐pL droplets and employs fluorescence microscopy combined with an immunoassay that detects fluorescence displacement on paramagnetic nanoparticles aligned in beadlines. In contrast to traditional approaches like ELISPOT, flow cytometry, CyTOF, and single‐cell sequencing, which primarily deliver endpoint data, DropMap enables real‐time quantitative measurement of protein secretion. Its applications include assessing TNF‐α secretion suppression in individual monocytes from septic shock patients, investigating immune responses by tracking cytokine secretion kinetics in single T cells, and determining antibody affinity from single B cells.

Lamanna et al. [117] introduced an innovative microfluidic system that enhanced the extraction of target cells from limited sample volumes, enabling the integration of single‐cell sequencing data with immunofluorescence‐based phenotypic profiling (Figure 9B). The platform combines digital microfluidics, laser‐induced cell lysis, and artificial intelligence‐driven image analysis to isolate individual cells from complex populations with precision. Following isolation, genomic and transcriptomic data are obtained through next‐generation sequencing, while proteomic analysis is conducted using nanoflow liquid chromatography coupled with tandem mass spectrometry. This study demonstrates sequencing performance that matches or exceeds existing advanced techniques, achieving single‐nucleotide resolution.

Xu et al. [118] introduced a novel digital microfluidics‐based method for single‐cell RNA sequencing (digital‐RNA‐seq), offering a streamlined, efficient, and cost‐effective solution for analyzing single‐cell mRNA (Figure 9C). The approach employs automated handling of discrete droplets to execute sequential scRNA‐seq protocols. To overcome limitations in single‐cell isolation such as low efficiency, compromised sample quality, poor selectivity, and limited flexibility, they propose a passive dispensing technique. This method uses precisely designed hydrophilic‐hydrophobic microstructures to quickly produce single‐cell subdroplets from cell suspension droplets. By utilizing nanoliter‐scale reaction volumes and hydrophobic interfaces, the system enhances gene detection sensitivity during cDNA synthesis and amplification. Additionally, stable droplet control and an oil‐sealed reaction environment ensure accurate mRNA quantification in digital‐RNA‐seq.

### 3.3 Pediatric Disease Treatments

Effective therapeutic intervention constitutes the fundamental cornerstone of pediatric care. However, the distinct physiological landscape of children—characterized by reduced blood volumes, heightened metabolic variability, ongoing maturation of organ systems, and disease phenotypes unique to developmental stages—necessitates rigorously tailored approaches. These approaches must account for altered pharmacokinetics/pharmacodynamics, heightened susceptibility to iatrogenic injury, and evolving immune responses, thereby diverging substantially from adult therapeutic paradigms.

Microfluidic technology is revolutionizing pediatric diseases treatment by enabling minimally invasive and precisely targeted strategies. Notably, micro‐integrated blood purification system utilizes a micro‐separation mechanism to safely remove specific components from small volumes of children's blood samples, significantly enhancing the management of leukemia or poisoning [119, 120]. Concurrently, novel respiratory support platforms address neonatal emergencies, whereas the micromembrane oxygenator reduces cardiopulmonary bypass risks in vulnerable newborns. These advancements demonstrate the ability of microfluidics to overcome pediatric‐specific treatment barriers through miniaturization, enhanced biocompatibility, and physiological simulation, ultimately promoting more accessible and adaptive pediatric care.

#### 3.3.1 Separation Therapy

In pediatric patients, hematologic diseases such as acute leukemia and platelet disorders are prevalent, often necessitating blood component separation as a critical therapeutic approach. These diseases frequently involve abnormal leukocyte proliferation or dysfunctional platelet activity, leading to a need for precise removal or manipulation of these components to mitigate symptoms and support therapeutic interventions [121]. Traditional blood separation method, such as centrifugation, while effective, can be time‐consuming and require specialized equipment that may not be readily available in all clinical settings. In contrast, microfluidic devices offer a highly efficient alternative, capable of manipulating fluid flow at the microscale using intricate microchannels and uniquely engineered geometries to achieve rapid and selective separation of blood components including leukocytes, platelets, and plasma [122, 123]. These devices leverage physical principles such as inertial focusing, dielectrophoretic, or deterministic lateral displacement to sort and isolate cellular components with high precision, minimizing the need for extensive sample preparation and large sample volumes—an essential consideration in pediatric care, where blood samples are often limited. Moreover, modern microfluidic platforms can be integrated with advanced diagnostic technologies, enabling real‐time detection and analysis of the separated components within the same device. This integrated approach not only streamlines the process of blood separation but also provides immediate feedback on the functional status of the isolated cells or plasma, thereby enhancing clinical decision‐making and treatment customization.

**FIGURE 10:**
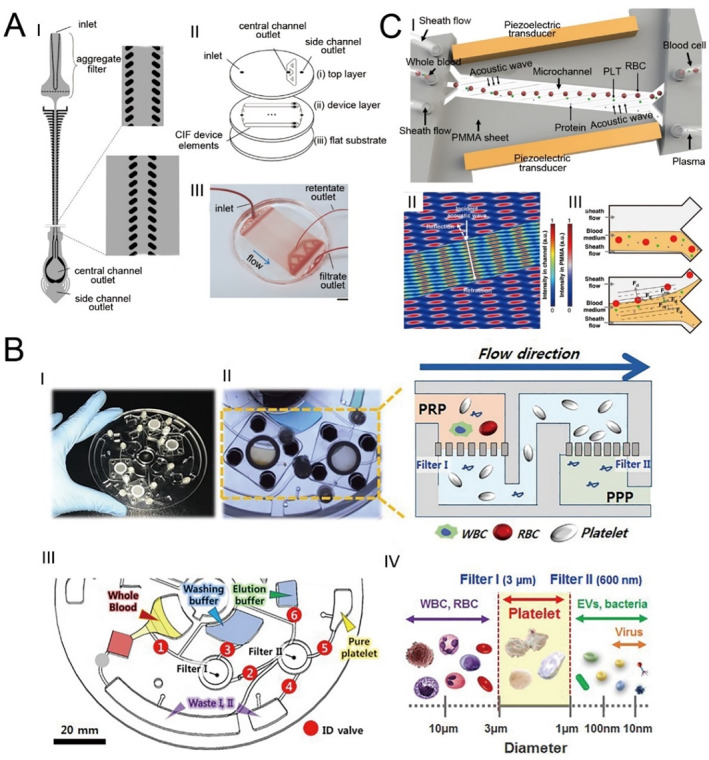
Microfluidic technology for separation therapy. (A) Design schematic and actual image of the CIF‐based microfluidic device. Reproduced under terms of the CC‐BY license [124]. Copyright 2022, The Authors, published by Springer Nature. (B) Process schematic of a fully automated, label‐free microfluidic device for platelet isolation. Reproduced with permission [125]. Copyright 2020, Royal Society of Chemistry. (C) Schematic of impedance mismatch‐assisted, tilted‐angle acoustofluidic plasma separation. Reproduced under terms of the CC‐BY license [126]. Copyright 2024, The Authors, published by Springer Nature.

Lezzar et al. [124] developed an innovative high‐throughput microfluidic device utilizing controlled incremental filtration (CIF) technology, featuring a void volume of 0.4 mL, as a potential alternative to centrifugation for leukapheresis (Figure 10A). This microfluidic system achieves leukocyte separation at nearly twice the speed of traditional centrifuge‐based leukapheresis and minimizes platelet loss by a factor of 2–3, highlighting its superior efficiency in blood component separation.

Recent studies suggest significant biological and functional differences between small and large platelets. Pediatric patients with immune thrombocytopenia (ITP) often require low‐volume, high‐quality platelets. Kim et al. [125] utilized a centrifugal microfluidic device to achieve fully automated platelet separation from blood (Figure 10B). Compared to traditional methods, this approach offers higher yield and purity, with lower platelet activation rates.

Ma et al. [126] introduced an acoustofluidic microfluidic system that efficiently eliminates red blood cells (RBCs), white blood cells (WBCs), and platelets from whole blood in a single operation (Figure 10C). By exploiting variations in acoustic impedance between different fluids, the platform generates forces on suspended particles that are substantially stronger than those produced by traditional microfluidic techniques. This capability enables the simultaneous removal of larger blood cells and smaller platelets in one step while preserving the integrity of proteins and antibodies present in the sample during the separation process. These technological advances improve the efficiency, speed, and safety of blood component separation, particularly in pediatric settings where traditional methods may be less feasible.

#### 3.3.2 Treatment for Pediatric Specific Diseases

Microfluidic technology demonstrates unique potential in the treatment of certain pediatric‐specific diseases, offering novel and effective therapeutic approaches for conditions affecting neonates and preterm infants, such as respiratory distress syndrome (RDS) and neonatal jaundice. This technology enables precise handling of small sample volumes, reduces treatment risks, and significantly improves therapeutic outcomes, positioning microfluidics as a promising option for enhancing prognoses in critically ill pediatric patients.

**FIGURE 11:**
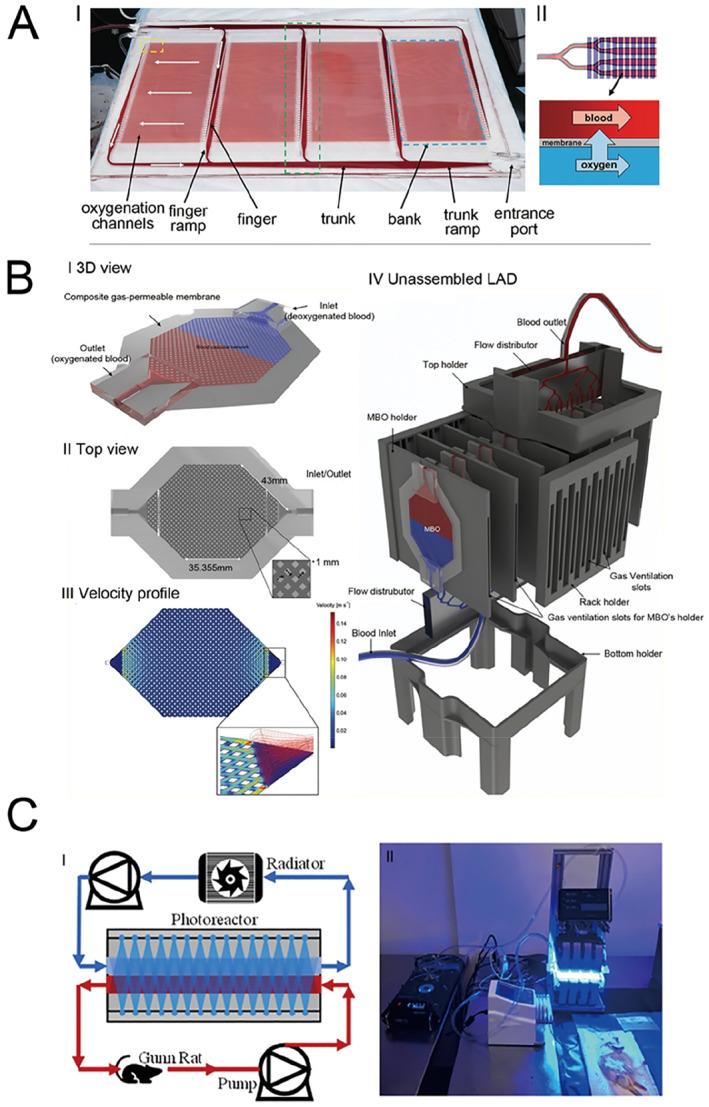
Microfluidic technology for treatment of pediatric specific diseases. (A) Overview of single layer of the BLOx device. Reproduced under terms of the CC‐BY license [128]. Copyright 2023, The Authors, published by Wiley‐VCH GmbH. (B) 3D schematic, top view, velocity profile, and unassembled layout of the MBO and LAD, illustrating uniform flow, air gaps, and bubble‐free blood movement. Reproduced under terms of the CC‐BY license [130]. Copyright 2020, The Authors, published by Wiley‐VCH GmbH. (C) Experimental setup for in vivo assessment of microfluidic photoreactor. Reproduced with permission [132]. Copyright 2021, The Authors, published by AIP Publishing.

Extracorporeal membrane oxygenation (ECMO treatment) is highly effective for critically ill children, but its use is limited by the complexity of the blood circuit, which can lead to coagulation and bleeding complications [127]. Recent studies have reported the development and clinical demonstration of the first microfluidic respiratory assist device (Figure 11A) [128]. This device features a fully three‐dimensional branched blood channel network that mimics key characteristics of physiological microcirculation, thereby avoiding the abnormal blood flow that causes thrombosis and hemolysis in traditional oxygenators. In 24‐h large animal experiments, the device demonstrated stable blood pressure reduction, low hemolysis, and consistent oxygen transfer, indicating its promising potential for broader applications.

Respiratory distress syndrome (RDS) is a common pathological condition in premature infants. It is primarily caused by inadequate surfactant production due to lung immaturity, leading to alveolar collapse, respiratory distress, and hypoxemia. Treatment for RDS typically involves administering artificial surfactant and mechanical ventilation [129]. A research team from Canada [130] successfully tested a microfluidic neonatal lung assist device (LAD) resembling an artificial placenta in a neonatal piglet model, which closely mimics the physiology of human preterm infants (Figure 11B). This LAD exhibited remarkable oxygenation efficiency using both ambient air and pure oxygen as sweep gases. Throughout the trials, the piglets experienced recurrent episodes of respiratory distress over prolonged durations, which were effectively mitigated by the LAD. These results indicate that the device holds promise as a bionic artificial placenta capable of supporting the respiratory requirements of preterm neonates.

Neonatal jaundice is a prevalent condition in newborns and is typically managed effectively through phototherapy [131]. In severe instances, however, exchange transfusion may become necessary. To provide an alternative to this invasive procedure, researchers have developed an ex vivo blood treatment employing a microfluidic photoreactor (Figure 11C) [132]. This innovative method utilizes the same underlying mechanism as phototherapy but incorporates microfluidic technology to enhance the rate of bilirubin removal. Animal studies have shown that this approach achieves reductions in blood bilirubin levels comparable to those attained with exchange transfusion.

## 4 Challenges and Prospects

### 4.1 Intrinsic Challenges in Microfluidic Technology

Despite its promising potential, microfluidic technology faces several challenges that hinder its widespread application and further development in pediatrics [20, 133]. One major obstacle is the complexity and interdisciplinary nature of microfluidic systems. The design and fabrication of microfluidic devices require expertise from diverse fields such as engineering, chemistry, biology, and materials science, which can present difficulties in communication, collaboration, and resource allocation [134]. Furthermore, the establishment of microfluidic systems, which involve the processing of chips and the assembly of fluid systems, presents significant challenges [135]. Although microfabrication techniques such as soft lithography, lithography, and micro‐milling have advanced considerably, producing microfluidic devices with high precision and reproducibility is time‐consuming and costly, especially for researchers lacking cleanroom facilities and manufacturing expertise [136, 137]. Moreover, scaling microfluidic systems remains a considerable hurdle. While microfluidic devices excel at handling small fluid volumes with high precision, scaling these systems to accommodate larger volumes or achieve higher throughput is problematic. Challenges such as uneven fluid flow, clogging, and increased variability between devices become more pronounced as the scale increases, limiting the scalability of microfluidics for certain applications [138]. Besides, integrating functional components such as sensors, actuators, and detectors into microfluidic systems introduces additional difficulties related to compatibility, miniaturization, and performance. In addition to the above problems, standardization and reproducibility are critical issues in microfluidics. Variability in fabrication processes, materials, and operating conditions can result in inconsistent outcomes, hindering the comparability of study results [139, 140, 141]. Therefore, establishing standardized protocols, quality control measures, and benchmarks for microfluidic devices is essential to ensure reproducibility and reliability for research and practical applications [41, 142].

### 4.2 Extrinsic Challenges in Pediatric Microfluidic Applications

In the field of pediatric medicine, the integration of microfluidics holds immense promise for revolutionizing diagnosis, treatment, and understanding of pediatric diseases. However, there are numerous challenges associated with applying microfluidics specifically in the field of pediatrics. One of the primary challenges lies in accurately replicating the intricate physiological dynamics of pediatric patients within microfluidic systems. Child development changes rapidly, with changes in organ size, function, and metabolic rate, so it is not easy to design microfluidic platforms that can accurately simulate these dynamic physiological states, necessitating rigorous age‐stratified validation protocols to ensure device performance and relevance from neonates to adolescents [143]. Achieving this level of fidelity requires a deep understanding of pediatric physiology, coupled with advanced engineering techniques. Another significant hurdle is the limited availability of pediatric‐specific biomaterials and cell lines. Unlike adult populations, pediatric samples are often scarce, particularly for rare diseases or conditions. Furthermore, the ethics surrounding pediatric research require careful consideration of informed consent, privacy concerns, and the use of pediatric samples in research [144]. The need to balance scientific progress with ethical responsibility adds complexity to the development and validation of pediatric microfluidic devices. Additionally, devices intended for direct patient use or sample collection must adhere to stringent biocompatibility standards tailored for children's sensitive tissues and potential long‐term exposure, which often exceed requirements for adults [145]. The other problem is to shrink the microfluidic system to fit the small sample size of pediatric patients while maintaining accuracy and reliability. Miniaturizing components without compromising functionality requires more complex chip designs and precise manufacturing techniques. Finally, for applications involving home use or caregiver operation, such as point‐of‐care diagnostics or monitoring, the development of highly intuitive, user‐friendly interfaces is paramount [146]. Designing devices that are simple, robust, and safe for operation by parents or non‐specialist caregivers, potentially under stressful conditions, presents a unique engineering and human factors challenge. Overall, addressing these challenges is crucial to fully unlock the potential of microfluidics and advance its application in pediatric medicine.

### 4.3 Future Perspectives

Future advancements in microfluidic technology are expected to lead to greater complexity and precision, thereby facilitating its enhanced integration into pediatric healthcare. High‐precision microfluidic platforms will enable the detection of biomarkers at lower concentrations and the identification of disease characteristics with increased specificity, thereby improving early diagnostic capabilities. This progress will contribute to optimizing treatment outcomes and reducing the risk of adverse effects. Furthermore, the development of highly biocompatible microfluidic platforms will allow for the creation of customized microenvironments at the microscale, effectively simulating the physiological conditions of pediatric patients. Moreover, the synergy between microfluidics and emerging technologies such as artificial intelligence (AI), deep learning, and big data analytics holds the potential to transform pediatric diagnostics and treatment paradigms. The integration of AI algorithms with microfluidic systems will facilitate real‐time data analysis, enabling rapid interpretation of complex biological signals and automated decision‐making processes [147]. Deep learning techniques can be employed to identify intricate patterns within microfluidic assay data, thereby enhancing the predictive accuracy for disease progression and treatment responses [148]. Additionally, big data analytics can leverage the vast amounts of information generated from microfluidic experiments to uncover novel correlations and insights that traditional methods may overlook [149]. These technological integration will extend the capabilities of microfluidics beyond conventional laboratory applications, paving the way for more accessible, cost‐effective, and point‐of‐care diagnostics suitable for resource‐limited settings. As microfluidic technology continues to converge with AI, deep learning, and big data analytics, its role in pediatric medicine will expand, driving innovations that not only improve diagnostic precision but also fundamentally alter approaches to disease management and treatment.

Looking ahead, the potential of microfluidic technology to be applied in pediatric healthcare is extremely vast in the future. To further expand the application of microfluidic technology in pediatric diagnosis and treatment, more efforts should be made to further improve the performance of microfluidic platforms in all aspects. Collaboration between clinicians, technologists and researchers is essential to drive innovation and overcome existing challenges. By harnessing the power of microfluidics, we can aspire to transform pediatric healthcare, ultimately improving the lives of young patients and advancing the field of medicine.

## 5 Conclusion

This review summarizes microfluidic technologies in pediatrics, highlighting their advantages and limitations across diagnostics, research, and therapy. These systems enable rapid biomarker detection, sophisticated disease modeling, and novel treatments—demonstrating transformative potential for pediatric healthcare. Despite challenges in scalability, biocompatibility, and functional integration for clinical use, future progress will be driven by advances in materials science, automation, and emerging fields like AI and big data analytics. These innovations will boost the precision, efficiency, and accessibility of microfluidic platforms, accelerating their adoption in pediatric medicine. As the technology matures, it will increasingly address critical challenges in child health.

## Author Contributions

Xuting Zhang, Lexiang Zhang, and Changmin Shao conceived the review topic and framework. Extensive literature search, data synthesis, and analysis were the collective effort of Yanke Wang, Chao Niu, Xing Rong, Chang Jia, Jia Sun, Shiyang Song, and Lexiang Zhang. The manuscript was drafted by Xuting Zhang and Andong Liu, and then strictly reviewed, edited and approved by Changmin Shao. Fangfu Ye and Maoping Chu provided overall supervision and secured funding.

## Conflicts of Interest

Fangfu Ye is an associate editor for *Smart Medicine* and was not involved in the editorial review or the decision to publish this article. All authors declare that there are no competing interests.
